# How Do Roots Interact with Layered Soils?

**DOI:** 10.3390/jimaging8010005

**Published:** 2022-01-05

**Authors:** Nina Kemp, Vasileios Angelidakis, Saimir Luli, Sadegh Nadimi

**Affiliations:** 1 School of Engineering, Newcastle University, Newcastle upon Tyne NE1 7RU, UK; n.kemp1@ncl.ac.uk (N.K.); v.angelidakis2@ncl.ac.uk (V.A.); 2 Preclinical In Vivo Imaging Facility, Faculty of Medical Sciences, Newcastle University, Newcastle upon Tyne NE1 7RU, UK; saimir.luli@ncl.ac.uk

**Keywords:** particle-scale behaviour, micromechanics, soil fabric, computed tomography, deep learning

## Abstract

Vegetation alters soil fabric by providing biological reinforcement and enhancing the overall mechanical behaviour of slopes, thereby controlling shallow mass movement. To predict the behaviour of vegetated slopes, parameters representing the root system structure, such as root distribution, length, orientation and diameter, should be considered in slope stability models. This study quantifies the relationship between soil physical characteristics and root growth, giving special emphasis on (1) how roots influence the physical architecture of the surrounding soil structure and (2) how soil structure influences the root growth. A systematic experimental study is carried out using high-resolution X-ray micro-computed tomography (µCT) to observe the root behaviour in layered soil. In total, 2 samples are scanned over 15 days, enabling the acquisition of 10 sets of images. A machine learning algorithm for image segmentation is trained to act at 3 different training percentages, resulting in the processing of 30 sets of images, with the outcomes prompting a discussion on the size of the training data set. An automated in-house image processing algorithm is employed to quantify the void ratio and root volume ratio. This script enables post processing and image analysis of all 30 cases within few hours. This work investigates the effect of stratigraphy on root growth, along with the effect of image-segmentation parameters on soil constitutive properties.

## 1. Introduction

**Figure 1:**
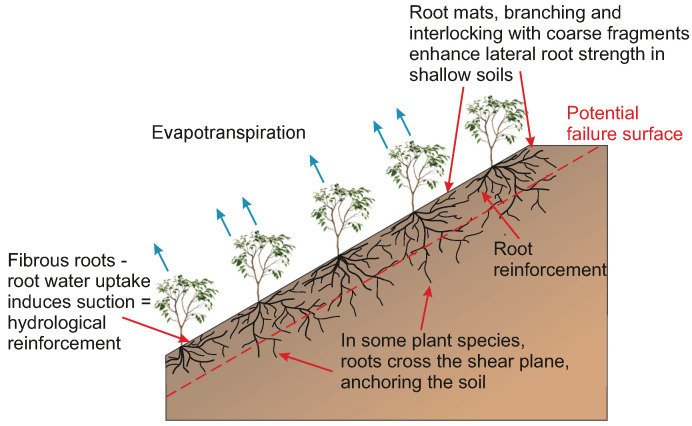
Root reinforcement in slope stability (inspired from [5]).

Roots are commonly employed as a bio-engineering technique for slope stability, particularly for infrastructure earthworks [1,2,3]. Plants stabilise the soil through mechanical reinforcement, as the root system causes an increase in the tensile strength of the soil [1,4]. This is most effective at shallow depths, where surface erosion and shallow slope failures occur [2] as seen in Figure 1. The root contribution to soil stability is governed by the root architecture and root mass, where shallow mat root systems are effective for erosion stability but are not suitable against shallow slope failures [5]. Stability is also provided through hydrological reinforcement, which results from evapotranspiration-induced matric suction increasing the shear strength of the soil, thus reinforcing the soil [1].

To aid in understanding the relationship between roots and soil for geotechnical engineering applications, the soil micro-structural changes induced by root growth must be quantified [6,7]. The influence of soil fabric on root growth has been extensively researched [8,9,10]. The soil fabric determines the strength and bulk density of the soil, which in turn governs the mechanical resistance of the soil. This establishes the response of a growing root, where roots grow into existing pores in the soil in order to avoid areas of high mechanical resistance [11]. Recently, there has been a growing interest in the influence of root growth on soil fabric. A major focus of this research is on whether root growth induces compaction in the immediate soil of the rhizosphere, or an increase in porosity [6,11,12,13]. X-ray micro-computed tomography (µCT) was employed by several research groups to reveal the micro-structure of geomaterials [14,15] and specifically to study soil–root interaction [12,16,17].

The aim of this paper is to understand how root growth alters the mechanical and physical properties of natural, layered soil. X-ray µCT is used to understand the behaviour of a growing root in two samples of a layered soil over 15 days. Image segmentation and deep learning allows the quantification of the soil mechanics parameter (i.e., the void ratio), and the root property (i.e., the root volume ratio). The importance of these parameters is evident in constitutive modelling [18,19,20,21], where these parameters contribute to the calculation of the soil strength, or quantifying the root reinforcement. These models allow the behaviour of soil to be predicted to assess the stability of slopes. In addition to supporting the engineering design of slopes and shallow landslides, this paper provides a methodology to process large volumes of imaging data efficiently using machine learning.

## 2. Materials and Methods

### 2.1. Sample Preparation

**Figure 2:**
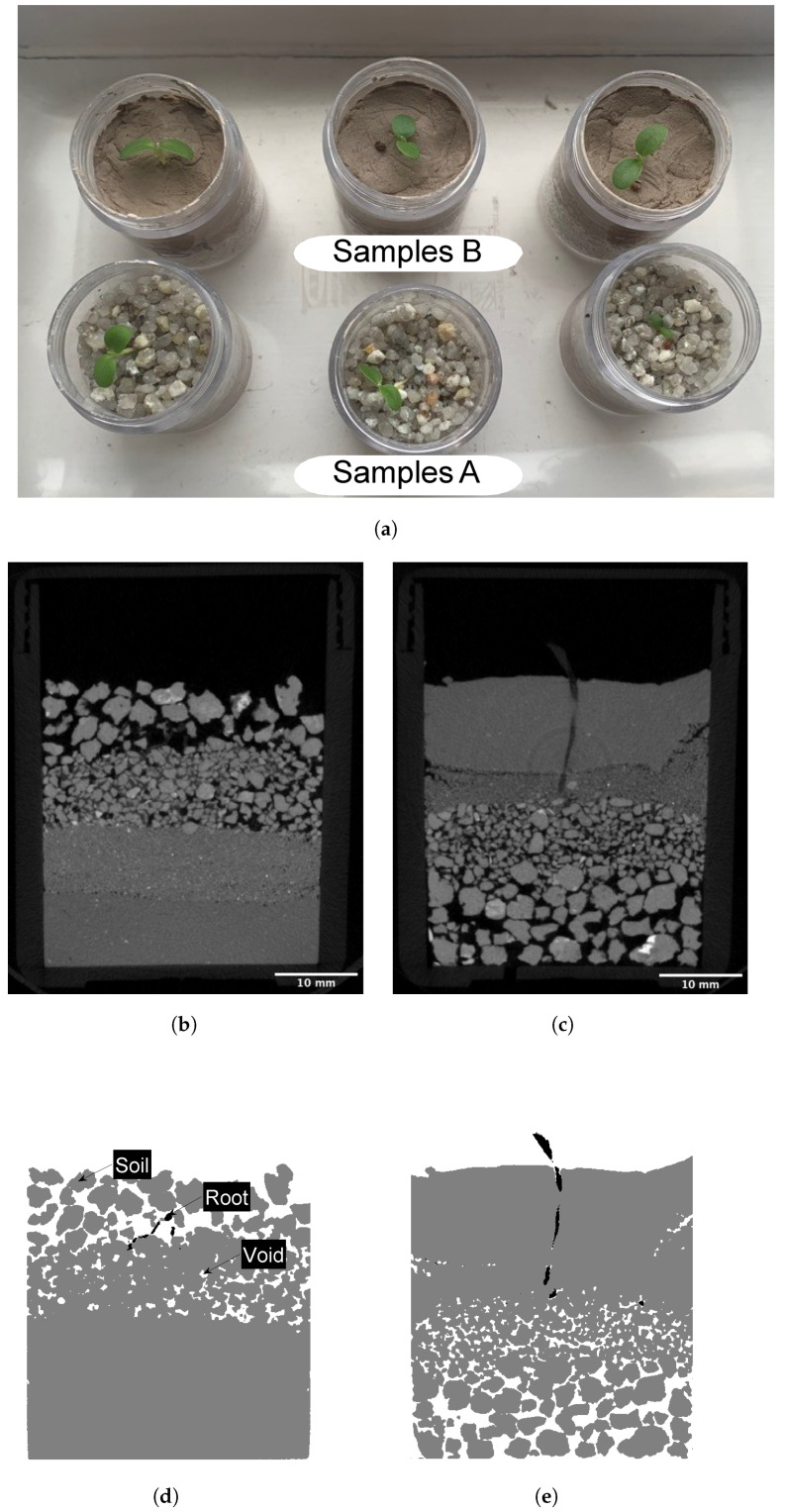
(**a**) Images of samples A and B on day 14 of the investigation, (**b**) µCT image reconstruction cross-section of sample A, day 15, (**c**) µCT image reconstruction cross-section of sample B, day 15. (**d**) labelled image of Sample A, (**e**) labelled image of Sample B.

Six cylindrical acrylic containers (32 mm inner diameter, 50 mm height, 3.2 mm wall thickness) are produced with two samples, A and B, for scanning. The remaining four samples are used for observation, as seen in Figure 2a. They are used to form a controlled sample, ensuring that root growth is not unduly affected by X-ray radiation. After 5 scans within 15 days of growth, no visual difference in the growth between the scanned and the controlled samples is observed. More accurate quantitative observation campaigns can be followed, measuring soil and biological quantities, such as the leaf index, water content and water uptake.

In vegetated slopes, soil is typically heterogeneous, and roots can migrate into soils with different gradation. Man-made embankments are constructed layer by layer with different grading depending on other engineering parameters. Studying the effect of the soil layering on root growth can be used to inform the design of vegetated slopes to better understand the root behaviour at soil interfaces. For sample A, the container is filled with ca. 1 cm clay at the base (soft to firm light brown clay of high plasticity − PI = 43), followed by ca. 1 cm silt, 1 cm sand and 1 cm gravel, whereas sample B consists of ca. 1 cm gravel at the base, followed by ca. 1 cm sand, 1 cm silt and 1 cm clay. Air pluviation of the silt, sand and gravel produces an unstructured homogeneous packing [6,22]. Seeds of *Achillea millefolium* germinate for 24 h before planting, and one seed is placed in each sample at a shallow depth in the centre of the container. This species is selected as the one used in engineering practice for soil stabilisation [23]. The samples are watered each day, ensuring watering is not carried out less than 24 h before scanning.

### 2.2. X-ray Micro-Computed Tomography

µCT is a non-invasive, non-destructive method that allows the 3D visualisation of root systems in situ in a soil column [24]. The µCT images obtained for this study are acquired using the SkyScan 1176 µCT system, located in the Preclinical In Vivo Imaging Facility at Newcastle University Medical School, United Kingdom. The µCT enables quantification of the void ratio and root volume during 15 days of growth. The acquisition and image reconstruction parameters are kept constant to enable automation in post-processing. This ensures that same materials are represented by voxels of the same intensity, allowing the training of the image classifier based on images from one sample and applying it to classify the images of other samples with same materials. The samples are scanned, with a source voltage of 90 kV and a current of 278 µA, on days 1, 4, 8, 11 and 15 after germination. Image reconstruction generates greyscale cross-sectional slices using the reconstruction algorithm from [25], with a pixel size of 35.2 µm, as seen in Figure 2b,c. The original images (dimensions 1360×1040×920) are re-sampled during post-processing to halve their size (680×520×460) for computational efficiency purposes, increasing the pixel size from 35.2 µm to 70.3 µm.

### 2.3. Image Segmentation

**Figure 3:**
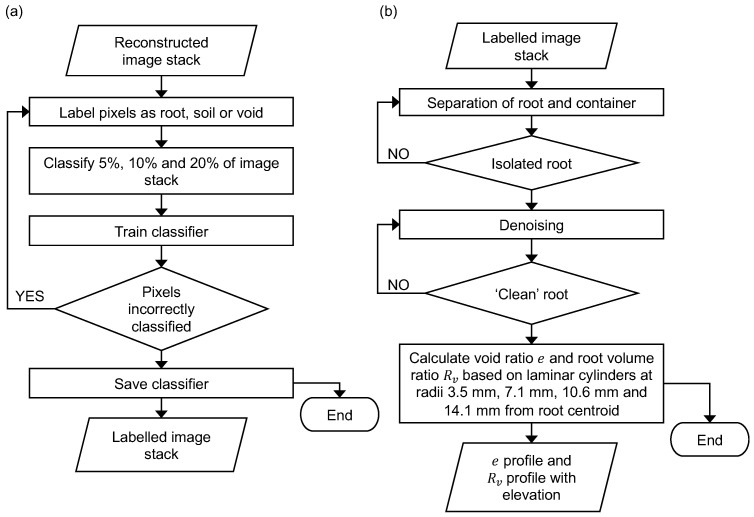
Flow charts representing the algorithmic steps of: (**a**) automatic classification process using deep learning (**b**) the MATLAB parameter quantification process.

Using the reconstructed images, the three phases of root, soil and voids are defined and identified using segmentation. The algorithmic steps of this process can be seen in Figure 3a. Segmentation is undertaken, using the open source software FIJI and the plug-in Trainable Weka Segmentation 3D [26]. The three classes of root, soil and voids are manually assigned to a set of input pixels of an image substack, with the classifiers developed based on training 5%, 10% and 20% of the image stacks. Each image stack representing one scan contains 680 re-sampled 2D slices of dimensions 520×460 pixels with 8-bit depth µm. Each classifier is trained and applied to all image substacks, creating labelled image stacks. The images used for training are selected to represent all three phases clearly. During supervised training, samples of each phase are identified to represent the range of intensity values corresponding to each of them. The exact operations of the machine learning algorithm used in this study are explained in [26]. The time taken to create and train a classifier is shown in Table 1. After labelling, small and isolated voxel clusters originally assigned to the root class (byproducts of segmentation) are removed during a noise elimination step, aimed at ensuring the root is identified accurately. In particular, isolated regions with fewer than 10 pixels are removed.

### 2.4. Quantifying the Void Ratio

**Figure 4:**
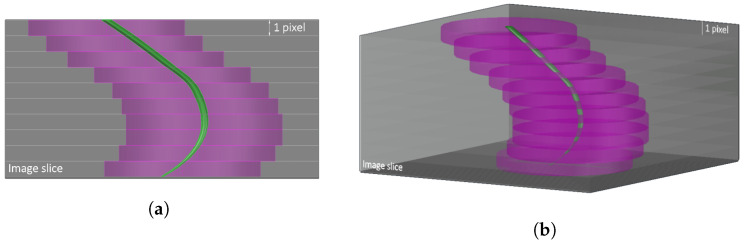
Schematic of laminar cylinder around root used as region of interest (ROI) to calculate parameters of geotechnical interest for varying elevation: (**a**) side view (**b**) perspective view.

**Figure 5:**
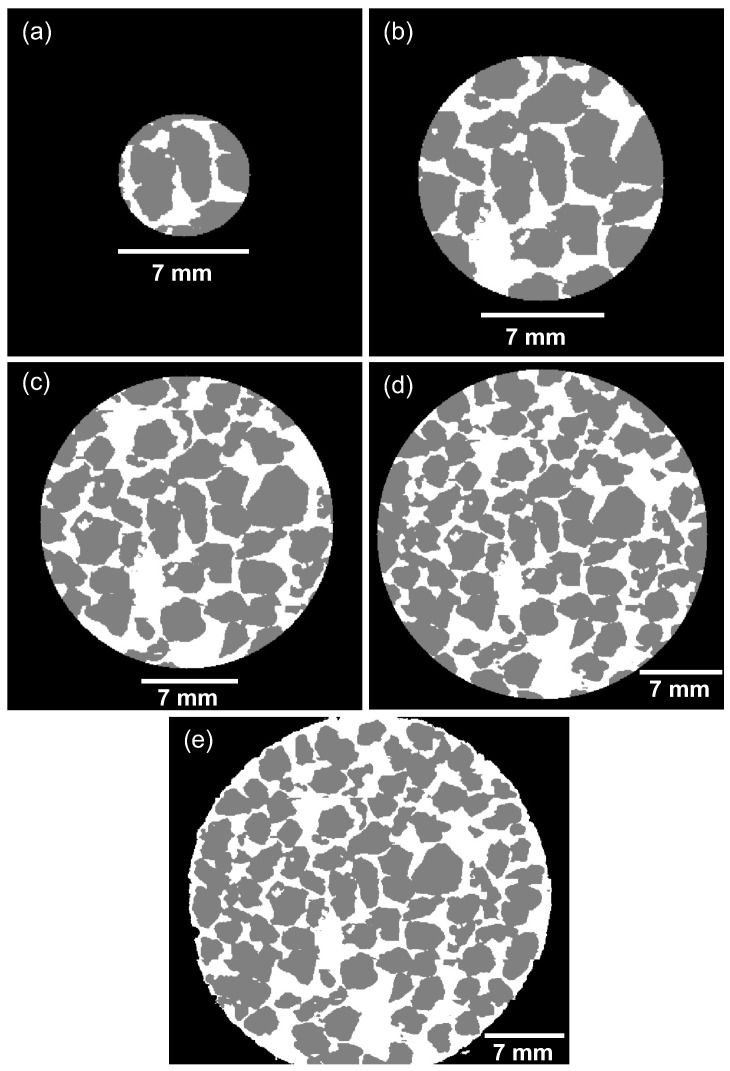
Sizes of the defined regions of interest: (**a**) radius of 3.5 mm, (**b**) radius of 7.1 mm, (**c**) radius of 10.6 mm, (**d**) radius of 14.1 mm, (**e**) whole container.

An automated MATLAB script is developed to perform parameter quantification, following the algorithmic steps seen in Figure 3b. Regions of interest (ROI) are defined, which are laminar cylinders in shape, as seen in Figure 4. These cylinders are defined at known distances around the root, moving along the length of the root. The void ratio is calculated as the total number of voxels representing the voids over the total number of voxels representing the solids, as seen in Equation (1). The local void ratio is quantified at cylinders with radii 3.5 mm (50 pixels), 7.1 mm (100 pixels), 10.6 mm (150 pixels), and 14.1 mm (200 pixels) from the centroid of the root, in addition to the global void ratio, as seen in Figure 5. Each section of the laminar cylinders is 2D, i.e., it has the width of 1 pixel size, as shown in Figure 4, while the radii of the various laminar cylinders considered in this study are visualised in Figure 5. These are calculated for each sample over 15 days, and using each classifier to determine the efficiency of the machine learning algorithm for calculating the soil mechanics parameters.

(1) $$e=\frac{\sum voxels_{void}}{\sum voxels_{solid}}$$

### 2.5. Quantifying the Root Volume Ratio

The root volume ratio is also quantified using the algorithm steps in Figure 3b. This root property is the total volume of roots per unit volume of soil, and is calculated as the total number of voxels representing the root over the total number of voxels representing the solids, as seen in Equation (2) [21]. This parameter is also calculated using the local and global ROIs to quantify the root and soil voxels. The global ROI corresponds to a calculation of the void ratio and root volume ratio considering the cross section of the whole container (32 mm), as shown in Figure 5e. The void ratio and root volume ratio are calculated for ROIs centred around a reference point found as the centroid of the the root pixels, for each 2D image slice. For slices with no root pixels, the void ratio is calculated considering each ROI centred around the centre of the container/image slice.

(2) $$R_{v}=\frac{\sum voxels_{root}}{\sum voxels_{solid}}$$

## 3. Results and Discussion

### 3.1. The Influence of Root Growth on Soil Fabric

**Figure 6:**
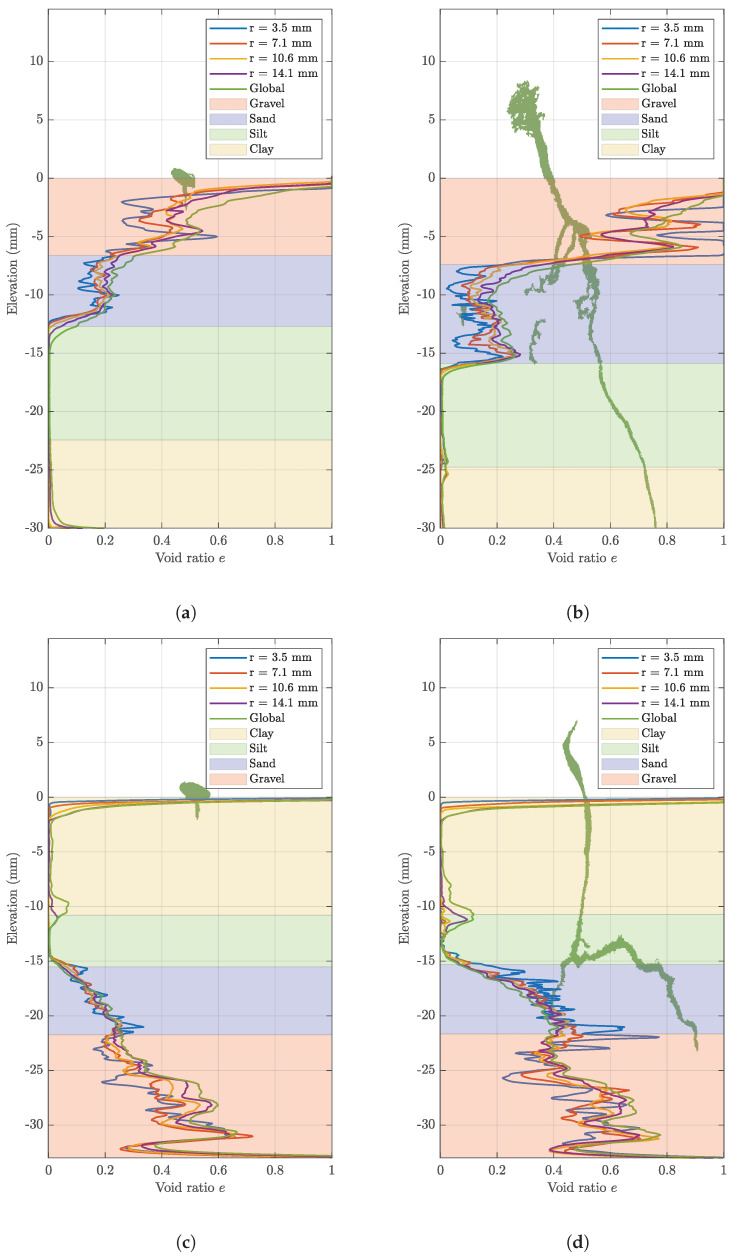
Void ratio profiles with elevation of (**a**) Sample A, day 1, (**b**) sample A, day 15, (**c**) sample B, day 1, and (**d**) sample B, day 15, using the 10% classifier (the layer thickness corresponds to averaged elevation for each stratum).

The void ratio is calculated with elevation in each sample, as seen in Figure 6. In both samples, the highest void ratio is found in the gravel layers, followed by the sand layers, with different trends observed with the distance from the root.

In sample A, the void ratio increases in the gravel layer from day 1 to day 15, as seen in Figure 6, with the highest void ratio in the immediate vicinity of the root, in the rhizosphere. At this distance, there is a large range in the void ratio with very high values reaching up to 1.0, whereas similar values of void ratio are obtained for the larger ROI sizes, as seen in Figure 6. This indicates that the void ratio quantified using the ROI with the radius of 3.5 mm may not be representative of the void ratio in the gravel layer. The larger ROI sizes are more likely to represent the void ratio of the soil, as with an increasing ROI size, the soil and root voxels increase proportionally. Therefore, the selection of these ROI sizes, as seen in Figure 5, is key to representing the void ratio in each sample accurately. In the gravel layer, the root induces an increase in the void ratio, suggested to be due to this fabric offering large voids that provide areas of low mechanical resistance [11]. Elongation of a root can result in the rearrangement and displacement of grains along its pathway [12,16,27]. It is suggested that the gravel offers the environment for this process to occur, resulting in an increased void ratio. The underlying sand layer experiences a decrease in void ratio, with the lowest void ratio observed closest to the root. This is attributed to the expanding root compressing the soil in the surrounding rhizosphere. This is supported by [28], who found that a growing root reduces the largest inter-aggregate pores by exerting stress that induces compaction. Extremely low values of void ratio are recorded in the silt and clay layers, due to the image resolution limiting the identification of the small pore spaces and, therefore, the root-induced changes in the rhizosphere. Local increases in the void ratio are observed in sample B on day 15 (Figure 2c) at the clay–silt interface near the container related to boundary effects.

Despite this, increases in the global void ratio at the clay–silt interface are evident in sample B (Figure 6). This can be attributed to cracking at this interface that is particularly evident at the container boundaries. Micro-crack formation can occur, as water uptake can cause localised variations in the water content, resulting in soil shrinkage [13]. When the root encounters the sand layer, an increase in the void ratio is induced. There is also a visible trend of a rising void ratio moving from the bulk soil into the rhizosphere, as seen in Figure 6. The change in soil fabric is proposed to have contributed to this increase, as the root moves from the silt layer to the sand layer where larger voids are present. This allows the root to displace sand grains for elongation, thereby inducing a higher void ratio in the root’s immediate vicinity. Although the root tip is visible in the top gravel layer by day 15, there is an increase in the void ratio where the root is present. This is observed for the ROI of 3.5 mm, where grain displacement likely occurs.

### 3.2. The Influence of Soil Fabric on Root Growth

**Figure 7:**
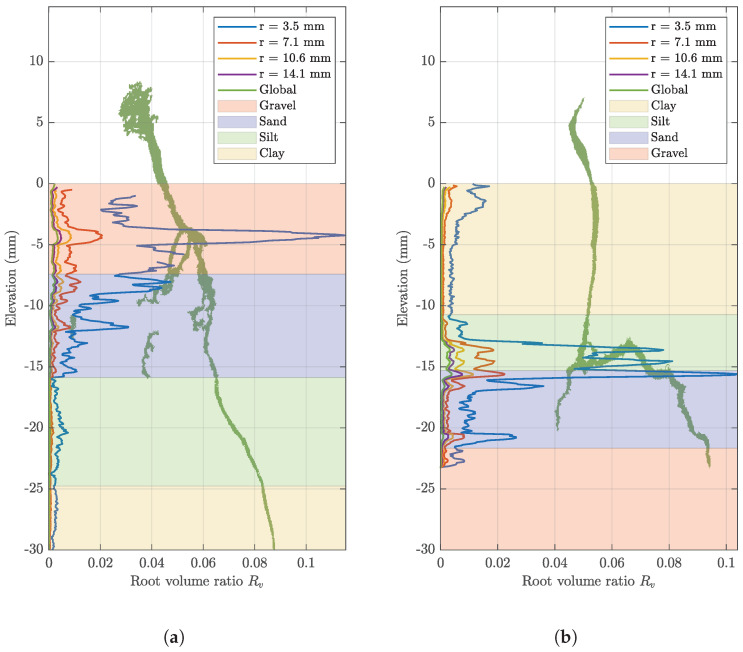
Root volume ratio profiles with elevation of (**a**) sample A, day 15 and (**b**) sample B, day 15, using the 10% classifier (the layer thickness corresponds to averaged elevation for each stratum).

The root volume ratio characterising each sample increases over time. For both samples, the root volume ratio decreases with distance from the root into the bulk soil, as seen in Figure 7. The sizes of the ROI also impact the root volume ratio profiles. As the size of the ROI increases, the number of root voxels remains constant, but the number of soil voxels increases. Increasing the size of the ROI results in a lower root volume ratio, thereby highlighting the significance of choosing a representative ROI that is able to quantify the impact of soil fabric on root growth meaningfully.

The gravel layer of sample A obtains the highest root volume ratio, with the ROI with the radius of 3.5 mm recording the highest root volume (Figure 7a). The thickest roots are also observed in this layer, highlighting the root response to this soil fabric. Roots generate an increase in diameter in response to a high mechanical resistance soil, as this limits the elongation rate [29,30]. Although the gravel contains large voids that the root will preferentially grow into to avoid areas of high mechanical resistance [11], the root also requires a large force to displace the large grains of gravel. This indicates that the root’s response to this layer is an increased diameter in order to displace grains and exploit the voids.

Once the root passes the gravel layer, the root volume ratio decreases with decreased elevation. The clay layer yields the lowest root volume ratio, as exhibited by a thin root. Image analysis enables the growth direction to be observed, with the root growing mainly vertically through the layers of sample A. It is observed that the root in sample A demonstrates a more complex structure in the coarser gravel and sand layers, where it grows around the soil grains, while it follows a more linear growth pattern in the finer silt and clay layers. The small grain size of fine soils allows the root to displace them, instead of growing around them (see Figure 7a).

Sample B demonstrates the greatest root volume at the silt–sand interface (Figure 7b). At this point, horizontal growth of the root occurs. The root re-orientates its growth upwards when encountering the sand interface, which is suggested to be due to the root facing an obstacle, such as a sand grain. The obstacle causes the root to alter its growth direction, as the root grows horizontally until meeting a void in the sand layer that it can exploit. The root then returns to its initial downward trajectory through the sand layer. This restriction to root elongation is also indicated by a visibly thicker root in the silt and sand layer, in contrast to the root characterising the clay layers. The root response to the gravel in sample B is not observed due to the root tip only being visible in this layer on day 15. It is observed that the root in sample B follows a simple, linear growth path in the finer clay and silt layers, and evolves to a more complex growth pattern when reaching the interface with the coarser sand layer (see Figure 7b).

It can be asserted that in both samples, the root structure exhibits more complex growth patterns when moving to areas of higher mechanical resistance (coarser soils), where roots need to grow around obstacles, and more linear growth patterns when moving to areas of lower mechanical resistance (finer soils).

### 3.3. Effectiveness of the Machine Learning Training Data Set Size

**Figure 8:**
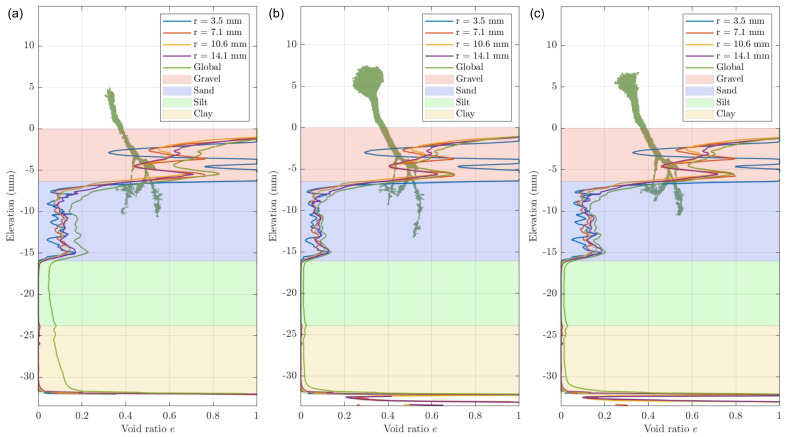
Void ratio profiles with elevation for sample A, day 11 using the classifier trained based on (**a**) 5%, (**b**) 10%, and (**c**) 20% of the image stacks (the layer thickness corresponds to averaged elevation for each stratum).

**Figure 9:**
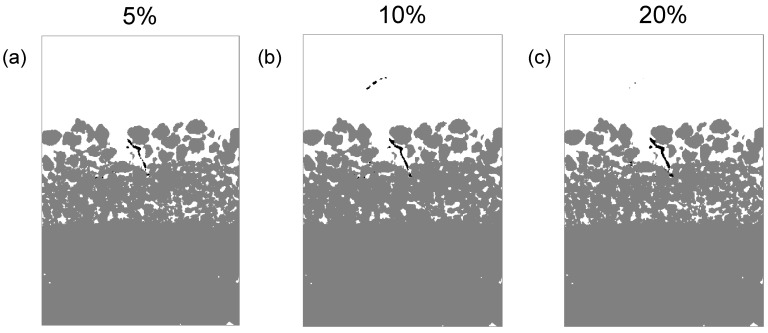
Segmented image slices of sample A, day 11 corresponding to the classifier trained based on (**a**) 5%, (**b**) 10%, and (**c**) 20% of the image stacks.

As the classifiers are trained to act at different training percentages, the outcomes illustrate the discrepancies in the accuracy of the segmentation. The results from each classifier display similar trends in void ratio and root volume ratio; however, the degree of change in the values varies. As expected, the 10% and 20% classifiers generate image labelling of higher quality in comparison to the 5% classifier (Figure 8). As seen in Figure 8b,c, the void ratio outcomes using the 10% and 20% classifier are similar. However, there are observable differences between these results, as the 10% classifier produces a lower void ratio in comparison to the 20% classifier. In Figure 8, there is also a marked difference in the volume of root identified across the different classifiers, as the 5% and 20% classifiers demonstrate that features of the root are lost. This suggests that the 10% classifier is able to obtain more accurate segmentation, as over-segmentation in the 20% classifier results in the root voxels being broken into smaller groups, whereas the 5% classifier produces poor results due to under-segmentation (Figure 9). This analysis suggests that in most cases, the 10% classifier obtains enhanced results, with marked differences in segmentation depending on the extent of training undertaken. The values of the void ratio and root volume ratio are provided as supplementary material for all samples, days of scanning and training data set sizes.

### 3.4. The Effect of ROI Size

A representative ROI size depends on the observed parameter of interest, as it can be different when measuring values of the void ratio or values of the root volume ratio. For the void ratio, the larger the ROI, the more probable it is to establish a representative area of the sample, as the number of pixels of solids and voids should increase proportionally once a representative ROI is established (see Figure 5). The same cannot be said for the root volume ratio, as the number of root voxels in each image is constant, while the volume of solids increases for ROIs of increasing radii. Thus, the larger the ROI, the smaller the root volume ratio values.

A rigorous calculation of a representative ROI size should relate to the grain size. Looking into the full range of results provided in the supplementary material, it becomes evident that the radius 14.1 mm (200 pixels) provides close void ratio values compared to the global ROI. Regarding the root volume ratio, the ROI size can be informed by the constitutive modellers, as their assumptions can vary.

## 4. Conclusions

In this paper, the use of µCT for observing the soil–root interaction was demonstrated, allowing root-induced changes in the rhizosphere and the bulk soil to be compared, along with the variations in root growth. These changes were quantified through the void ratio and the root volume ratio, with the importance of these parameters being evident in constitutive models. Segmentation was undertaken to classify the features of root, soil and voids using a machine learning algorithm along with deep learning to perform parameter quantification. The effectiveness of the machine learning algorithm technique was also examined for different sizes of training data sets, revealing that a greater amount of training does not necessarily lead to better classification of the materials of interest. Instead, user dependency is present, during the assignment of voxels to material classes, which are used to train the machine learning algorithm.

The outcomes highlighted the importance of the soil fabric and the distribution of stratigraphy on root growth. In most cases, the root growth resulted in an increase in the void ratio. However, root-induced compaction was also observed in the sand layer due to the expansion of the growing root. The influence of the soil fabric on the direction of root growth was also demonstrated, where the root’s response to encountering a sand grain was a large deviation in its growth trajectory. This paper highlighted the complex nature of the rhizosphere, with the root-induced changes in this zone being dependent upon the soil fabric. Further investigations using an enhanced image resolution should be undertaken to visualise changes in the clay and silt layers, along with the use of varying water contents to observe the effect of suction on changes in the rhizosphere. Additionally, possible future work includes reducing the user dependency of the machine-learning classifier, establishing a physics-based ROI size for geotechnical parameters and acquiring 3D images of soils from natural slopes. Understanding the changes at the soil–root interface and their implications aids the prediction of soil behaviour in geotechnical design.

## Figures and Tables

**Table 1:** Time taken to create and train the classifier.

Percentage of Images Used to Train Classifier	Time to Create Classifier (min)	Time to Train (min)
5%	38	14
10%	58.5	40
20%	128.5	42

## Data Availability

The image-analysis data presented in this study are openly available in data.ncl.ac.uk at 10.25405/data.ncl.16734565 (accessed on 5 October 2021) [31].
